# Effect of Different Artificial Staining Procedures on the Color Stability and Translucency of a Nano-Hybrid Resin-Based Composite

**DOI:** 10.3390/ma16062336

**Published:** 2023-03-14

**Authors:** Gaetano Paolone, Claudia Mazzitelli, Francesca Boggio, Lorenzo Breschi, Alessandro Vichi, Enrico Gherlone, Giuseppe Cantatore

**Affiliations:** 1 Dental School, IRCCS San Raffaele Hospital, Vita-Salute University, 20132 Milan, Italy; 2 Department of Biomedical and Neuromotor Sciences, DIBINEM, University of Bologna-Alma Mater Studiorum, Via San Vitale 59, 40125 Bologna, Italy; 3 Dental Academy, University of Portsmouth, William Beatty Building, Hampshire Terrace, Portsmouth PO1 2QG, UK

**Keywords:** color stability, color change, brushing, staining, resin-based composites, RDA, translucency, translucency parameter, contrast ratio

## Abstract

Background: To evaluate the effect of different experimental staining procedures on color stability and translucency of a nano-hybrid resin-based composite (RBC). Methods: Forty-eight cylindrical-shaped specimens (10 × 2 mm) were prepared with a nano-hybrid RBC (Clearfil Majesty ES-2) and randomly divided in four groups according to the experimental staining procedure: G1) static immersion in a staining solution (coffee) (44 ± 1 °C); G2) staining cycling between coffee (44 ± 1°C) and distilled water (37 ± 1°C) with an experimental staining machine based on Arduino, an Open Source hardware development platform; G3) staining cycles as in G2 + brushing with a low abrasive toothpaste (Relative Dentin Abrasion RDA = 30) (Elmex Sensitive Professional); G4) staining cycles as in G3, with brushing performed with a very strong abrasive toothpaste (RDA = 90) (Lacult Active). Color parameters were recorded at the baseline (T0) after staining procedures (T1) and repolishing (T2) using a spectrophotometer. Color change (∆E00) and translucency (TP, CR) were evaluated. Data were statistically analyzed (*p* < 0.05). Results: For ∆E00 after staining, Group 1 showed the highest color change and Group 3 the lowest. All groups were significantly different (*p* < 0.001) except for Group 2 vs. Group 4; after repolishing, Group 1 was significantly higher than Group 3 (*p* < 0.001), Group 2 (*p* < 0.001), and Group 4 (*p* = 0.003); Group 2 was higher than Group 3 (*p* < 0.001). For TP variable, after staining procedures, Group 2 was significantly higher than all other groups (*p* < 0.001), and Group 1 was significantly higher than Group 3 (*p* < 0.001) and Group 4 (*p* = 0.007). After repolishing, Group 4 was significantly lower than Group 3 (*p* = 0.008) and Group 2 (*p* = 0.027). Repolishing procedure significantly reduced color parameters. Conclusions: The investigated staining procedure induced significant differences in color stability and translucency. The use of a very strong abrasive toothpaste (RDA = 90) induced higher color change than a low abrasive one (RDA = 30). Repolishing procedures are able to partially reduce color change induced by artificial staining procedures.

## 1. Introduction

Despite significant technological advancements, the stability of the optical properties (color stability and translucency) of resin-based composites (RBCs) in the oral environment remains a persistent issue [1]. Changes in RBCs’ optical properties depend on the different characteristics of the oral environment, often characterized by abrasive, erosive, and staining challenges [2,3]. Several studies have reported that when resin composites come into contact with staining agents, they absorb extrinsic pigments responsible for color change [1], irrespective of whether they are intended to be used for direct or indirect restorations [4]. This effect is also increased when RBCs are exposed to alcoholic and acidic media due to the degradation of the organic matrix [5]. Nevertheless, complete knowledge of the complex interactions between the factors that occur in the oral environment and are responsible for the material’s color instability still needs to be achieved.

Furthermore, involved variables are challenging in recreating in an in vitro experimental design, resulting in conflicting scientific evidence [6,7]. In many papers investigating color stability, staining procedures are generally performed by a simple static immersion of RBC specimens in staining media [1]. Few papers have used more complex in vitro simulations including specific temperatures, cycling between staining and neutral solutions (water or artificial saliva) [8], smoking [9,10,11], pH variations [3], or tooth brushing simulation [12,13,14,15].

In a recent review [1], only 8 out of 178 papers used different temperatures according to the solutions used (e.g., warmer for hot beverages such as coffee, colder for soft drinks), and only 2 used a cycling device between different liquids and temperatures. Regarding the tooth brushing simulation, it was rarely used [8,13,14,16,17]. Previous studies have reported the benefit of brushing in reducing the staining of RBCs; [8] however, at the same time, brushing could be responsible for the increase in the surface roughness [12]. The extent to which brushing reduces extrinsic pigmentation or increases roughness depends on various factors, such as the characteristics of RBCs matrix and filler particles, the cumulative brushing time, and the abrasiveness of the toothpaste [12,15]. RDA stands for “Relative Dentin Abrasion,” which is a measure of toothpaste’s abrasiveness. This value is determined by laboratory testing and is expressed as a number on a scale. A lower RDA value indicates a less abrasive toothpaste, which is generally considered better for oral health. Imfeld et al. [18] classified toothpaste based on RDA values: very low abrasion = <20; low abrasion = 20–40; moderate abrasion = 41–60; strong abrasion = 61–80; very strong abrasion = >80. It has been reported that high abrasion may result in an increase in surface roughness and a decrease in polishing; in general, a low abrasion toothpaste should be preferred [19,20,21]. The potential increase in surface roughness and a decrease in polishing can impact the appearance of the composite and potentially contribute to the accumulation of plaque, ultimately leading to the failure of the restoration [22,23]. Although it is clear that many factors contribute to the degradation and discoloration of RBCs, a truthful experimental design for RBC staining procedures is far from being defined.

Therefore, this study aimed to determine the effect of different experimental staining procedures (dynamic staining and brushing) on the optical properties (color change and translucency) of a nano-hybrid RBC. In particular, the null hypothesis tested were that the color stability and translucency are not influenced by (1) the staining procedures; (2) the toothpaste; and (3) the repolishing.

## 2. Materials and Methods

One nano-hybrid RBC (Clearfil Majesty Es-2 Classic A3, Kuraray Noritake Dental Inc., Tokyo, Japan) was used for the specimen’s fabrication. Characteristics of the investigated resin-based composite is described in Table 1.

Forty-eight specimens were fabricated using a Teflon mold with a central hole of a 10 mm diameter and a 2 mm thickness positioned over a glass plate and a polyester strip. The resin composite was placed in one increment into the mold and was covered by another polyester strip and glass plate. An axial load of 500 g was applied for 60 s to promote smoothness and to remove the excess resin composite. The specimen was then photoactivated for 20 s through the glass plate, and additionally 20 s after removing the plate, using a light-emitting diode (LED) light source (VALO, Ultradent Products, South Jordan, UT, USA) with 1000 mW/cm^2^ for 20 s. Specimens were polished with a series of polishing discs (Sof-Lex medium, fine, superfine, 3 M ESPE, St. Paul, MN, USA) with a handpiece at 15,000 rpm for 5 s for each disc. Discs were changed every specimen. The bottom surface as well as the lateral one of all the cylindric specimens were coated with a transparent nail varnish (Classic Nail Enamel, Clear, Revlon, New York, NY, USA) leaving the top surface uncovered. The specimens had their thickness measured with a digital caliper (Absolute Digimatic, Mitutoyo, Tokyo, Japan). Specimens that varied more than 0.05 mm from the ideal (2 mm) thickness were discarded. Specimens that fit the thickness criteria were then stored in distilled water for 24 h at 37 °C to allow post-curing. The roughness of each disc has been checked with a surface profilometer (Mitutoyo SJ-201P, Mitutoyo, Kanagawa, Japan) set with a cutoff value of 0.8 mm, a stylus speed of 0.5 mm/s, and a tracking length of 5.0 mm to check uniformity in Ra values among the discs.

Color measurements

Color coordinates, L*, a*, b* of the CIELab color system were obtained using a digital spectrophotometer (Vita Easyshade, Vita Zahnfabrik, Bad Säckingen, Germany) on a gray, a black, and a white background. At the beginning and after each group measurements, calibration was performed as indicated by manufacturer. All measurements were performed by a single trained operator with standardized D65 light room illumination. Three readings were performed for each specimen, and every background and the mean values of color coordinates were obtained. The color measurements were performed at baseline (T0), after staining procedure (T1), and after repolishing procedures (T2).

Color differences

To evaluate color differences between time intervals, the CIEDE2000 (∆E00) [24] color difference formula was used as follows:

$$\text{Δ}E00={\left[{\left(\frac{\Delta L^{\prime }}{K_{L}S_{L}}\right)}^{2}+{\left(\frac{\Delta C^{\prime }}{K_{C}S_{C}}\right)}^{2}+{\left(\frac{\Delta H^{\prime }}{K_{H}S_{H}}\right)}^{2}+R_{T}{\left(\frac{\Delta C^{\prime }}{K_{C}S_{C}}\right)}^{2}{\left(\frac{\Delta H^{\prime }}{K_{H}S_{H}}\right)}^{2}\right]}^{1/2}$$

where Δ*L*′, Δ*C*′, and Δ*H*′ are the differences in lightness, chroma, and hue for a pair of specimens using CIEDE2000. 𝑆𝐿, 𝑆𝐶, and 𝑆𝐻 are weighting functions for the adjustment of the total color difference for variation in perceived magnitude with variation in the location of the color coordinate difference between two color measurements. Parametric factors KL, KC, and KH in CIEDE2000 formula were set to 1.

Color differences were also evaluated through comparisons with 50:50% perceptibility (PT) and 50:50% acceptability (AT) thresholds. Considered PT and AT values for CIEDE2000 (1:1:1) were, respectively, ΔE_00_ = 0.81 and ΔE_00_ = 1.77 [25].

Translucency

The specimens’ translucency was calculated with the translucency parameter (TP) and contrast ratio (CR). TP was calculated using the following formula:

$$\mathrm{TP}=\sqrt[{2}]{{\left(L^{*}{}_{B}-L^{*}{}_{W}\right)}^{2}+{\left(a^{*}{}_{B}-a^{*}{}_{W}\right)}^{2}+{\left(b^{*}{}_{B}-b^{*}{}_{W}\right)}^{2}}$$

where the *W* refers to CIELab values on a white background while “*B*” on black background

The L* coordinates values measured on white and black background were also used to calculate the luminance from Color Space CIEXYZ as follows:

$$Y={\left(\frac{L+16}{116}\right)}^{3}\times Y_{n}$$

*Y* values of the specimens recorded on white (YW) and black (YB) backgrounds were used to calculate Contrast Ratio (CR) as follows:

$$\mathrm{CR}=\frac{\mathrm{YB}}{\mathrm{YW}}$$

TP and CR were calculated at t0, t1, and t2.

Staining solution

To prepare the staining solution, 24 gr of coffee powder (Nescafé Classic, Nestlé Italia, Assago, Italy) was poured into 2 L of boiling distilled water. After 10 min of stirring, the coffee solution was filtered through filter paper. The staining solution was kept at 44 ± 1 °C. Specimens were immersed in the staining solution for a total of 24 h corresponding to a consumption of 1 month [26] but at different intervals as specified below. The staining solution was renewed every 6 h.

Staining procedures

The specimens were randomly divided in four groups (*n* = 12) according to the staining simulation (T1) provided:

G1 (control): Specimens were immersed in 500 mL of staining solution at 44 ± 1 °C for 24 h.

**Figure 1:**
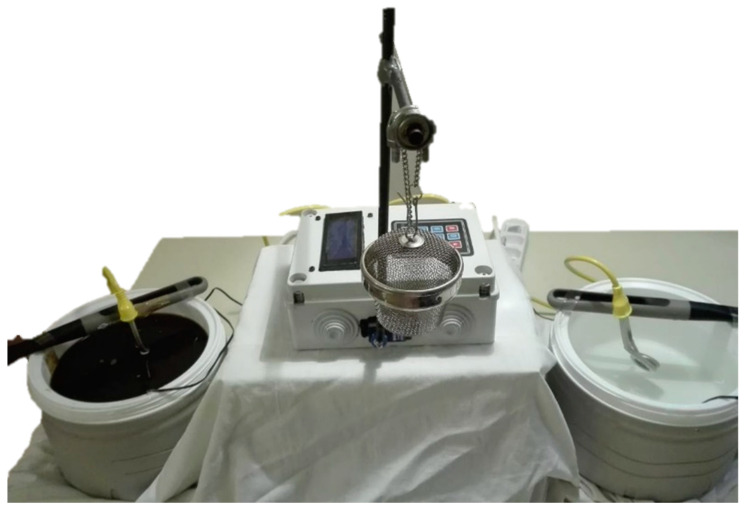
The experimental staining machine.

G2: Specimens were cycled through an experimental staining machine (Figure 1) based on Arduino^®^, an open-source development hardware (Arduino, Ivrea, Italy) programmed to move a robotic arm holding specimens. Specimens were cycled between the coffee staining solution (44 ± 1 °C) and distilled water (37 ± 1 °C) with a dwell time of 45 min and a transition time of 30 sec. Thirty-two cycles were performed, which corresponds to a total of 24 h immersion in the staining solution.

**Figure 2:**
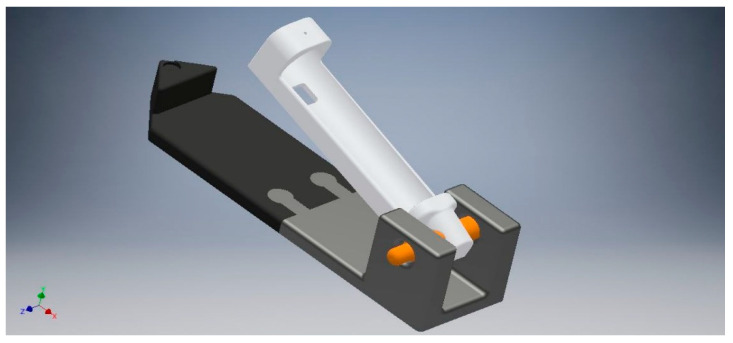
The 3D rendering of the experimental brushing machine.

**Figure 3:**
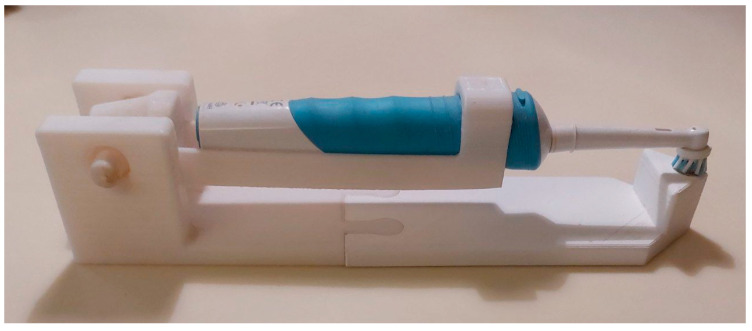
The 3D-printed experimental brushing machine.

G3: Specimens were treated as in G2 plus adding, after every immersion in the staining solution, a brushing simulation (7.5 sec) with an experimental brushing machine. The prototype, designed in 3D Autodesk Inventor (Autodesk, Mill Valley, CA, USA) (Figure 2), was 3D printed in Polylactic Acid (PLA) and designed to carry an electric toothbrush (Oral-B Vitality 2D Sensitive Clean, Procter & Gamble, OH, USA) (Figure 3) with a load of 200 g. Brushing procedures were performed with a low abrasive toothpaste (RDA = 30) (Elmex Sensitive Professional, Colgate-Palmolive, Anzio, Italy).

G4: Specimens were prepared as in G3 but with a very strong abrasive toothpaste (RDA = 90) (Lacult Active, Theiss Naturwaren GmbH, Homburg, Germany).

Repolishing

After staining procedures, each specimen was repolished (T2) using the same polishing procedure previously described for specimen fabrication.

Statistical analysis

Quantitative data were presented as mean and standard deviation unless otherwise specified. Shapiro Wilk test and graphical methods were used to check normality model assumptions. Linear mixed models were used to evaluate TP and CR parameters during the time points in the four groups. Time, groups, their interaction, and baseline values were the fixed factors, while specimens were the random factor.

The ΔE00 was evaluated with a linear mixed model where time, groups and their interaction were the fixed factors, while specimens were the random factor.

Estimated means and standard error (SE) were reported for each time and group. Comparisons between groups were performed at T1 and T2, and the Benjamini–Hochberg (BH) was applied for multiple comparison correction. Stata 16.1 was used for all analysis except the BH, for which R RStudio 2022.12.0 was used. A *p* value < 0.05 was considered statistically significant.

**Figure 4:**
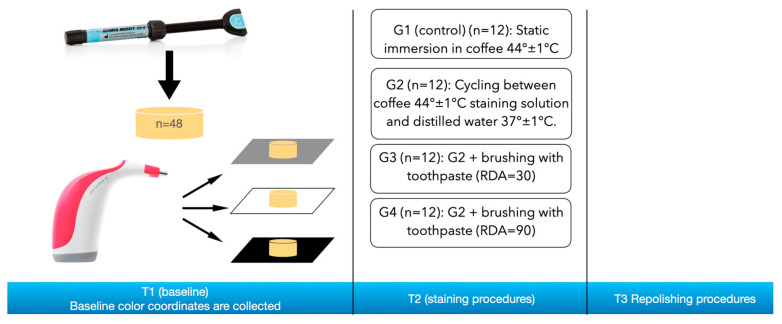
Graphical representation of the experimental design.

Figure 4 shows a graphical representation of the experimental design.

## 3. Results

Analysis was made on 48 samples in 4 groups and 3 timepoints.

Table 2 shows the values of TP and CR at baseline.

### 3.1. TP Parameter

**Figure 5:**
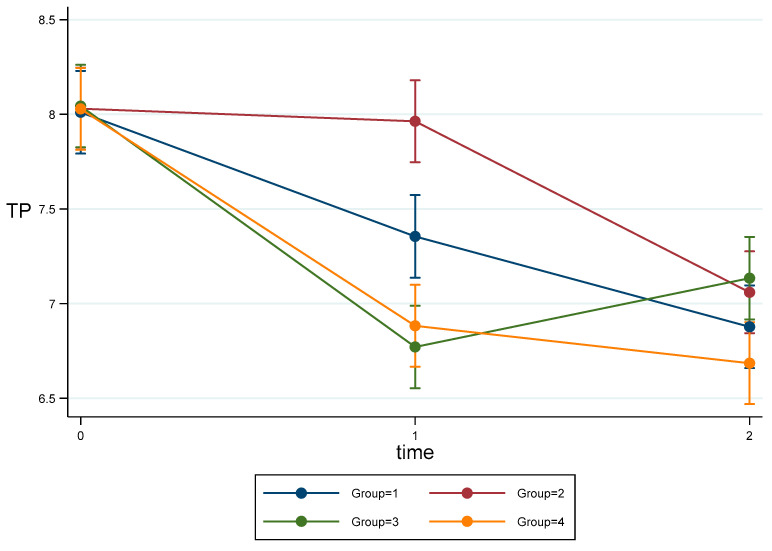
Estimated means and CI95% for TP Parameter adjusted for baseline values.

Time and interactions were statistically significant. Data at baseline did not show statistically significant differences. In the model adjusted for baseline values (Table 3, Figure 5) after BH correction, G2 was significantly higher than all others (*p* < 0.001) at T1, and G1 was significantly higher than G3 (*p* < 0.001) and G4 (*p* = 0.007). At T2, G4 was significantly lower than G3 (*p* = 0.008) and G2 (*p* = 0.027).

### 3.2. CR Parameter

Table 4 shows estimated means for each group and timepoint. Changes between T1 and T0 and T2 and T0 were significant in each group, with the exception of groups 1 and 2 in between T0 and T1 (respectively, *p* = 0.066; *p* = 0.738).

**Figure 6:**
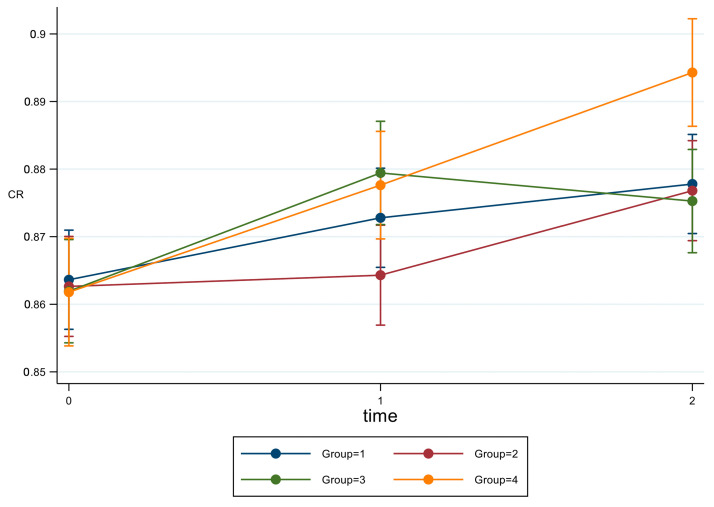
Estimated means and CI 95% from mixed model adjusted for baseline for parameter CR.

After BH correction (Figure 6) at T1, G2 was significantly lower than G3 (*p* = 0.015) and G4 (*p* = 0.038), while at T2, G4 was significantly higher than other all groups (*p* = 0.012).

### 3.3. *ΔE00* Parameter

**Figure 7:**
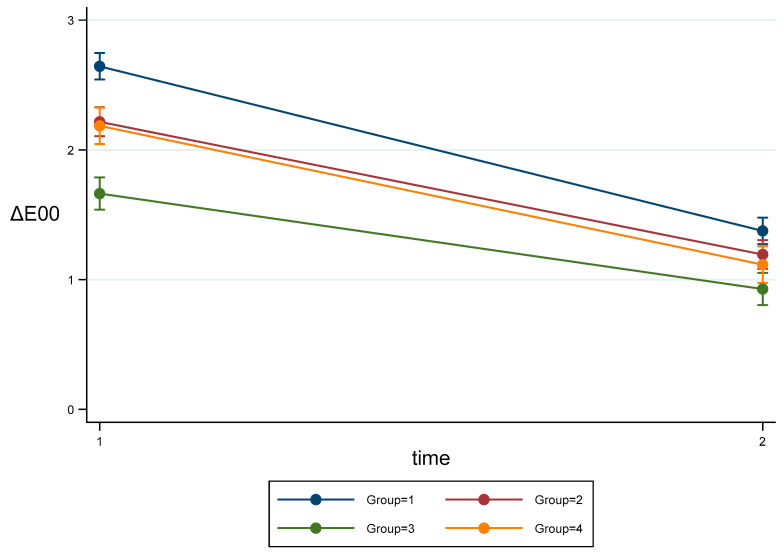
Estimated means and CI95% for ΔE00 Parameter.

Table 5 shows estimated means for each group and timepoint. Figure 7 shows mean values and their 95% CI. After BH correction, all groups were significant different (*p* < 0.001) except for Group 2 vs. Group 4. At T2 group, Group 1 was significantly higher than Group 2 (*p* = 0.024), Group 3 (*p* < 0.001), and Group 4 (*p* = 0.005), and Group 2 was higher than group 3 (*p* = 0.003).

## 4. Discussion

The growing significance of dental aesthetics in people’s professional and social lives requires restorative materials that can effectively mimic and replace the features of dental tissues lost due to caries or trauma [27,28,29]. In addition to providing improved mechanical properties, the restorations performed with these materials should simulate aesthetic characteristics, such as surface smoothness, color, translucency, and gloss of dental tissue, and maintain stability over time [27]. RBCs are in fact subject to exposure to extrinsic and intrinsic coloring agents that can influence color stability and translucency over time [1,9]. In the present study, significant differences were reported for ΔE_00_, TP, and CR values after staining (T1), leading to the conclusions that the investigated staining procedures significantly influences color and translucency. The first null hypothesis has therefore to be rejected. In order to investigate color changes over time, several studies leave specimens statically immersed in containers at room temperature [1]. In our study design, in order to become as close as possible to clinical conditions, variables were considered as follows: (1) the temperature (44 ± 1 °C) close to the one the staining solution is generally consumed at; (2) cycling between the staining solution (44 ± 1 °C) and distilled water (37 ± 1 °C), thus simulating a buffering effect and a discontinuous contact with the staining solution; (3) a brushing simulation using two kinds of toothpaste with different abrasive levels: low abrasion and very strong abrasion. The results show that static staining (G1) is responsible for the highest color change. Since the other three groups, characterized by conditions more similar to a real clinical scenario, show less color change, it can be confirmed that static immersion is responsible for an overestimated color change. Actual staining in the mouth would, in fact, be affected by discontinuous exposure to the stain, the dilution and buffer effect of the staining agent by saliva and other fluids, the mechanical contact with soft tissues (cheeks and lips), and the polishing of the restorations through toothbrushing [12].

Furthermore, a non-static staining procedure, such as the ones performed in G2, G3, and G4, may also prevent deeper penetration of the staining molecules inside the “bulk” of the material [7]. Staining, in fact, is caused not only by a deposition of the pigments on the surface (adsorption), but also by absorption: the penetration within the first layer of the material [30]. A prior study estimated that extrinsic pigment molecules could penetrate up to 3 to 5 μm in resin composites after seven days of coffee staining [31]. The effectiveness in reducing color change of resin composites is in fact dependent on the depth of pigment molecule penetration into the material [32].

The cycling between distilled water and the staining solution in G2, G3, and G4, produced a significantly lower color change, showing that interspersing a neutral solution such as water or saliva could somehow reduce the deposition of the pigments on the surface or the penetration inside the material. Nevertheless, neutral solutions such as water or saliva also may induce color change over time, but to a lower degree with respect to colored solutions, and these color changes can be attributed to the hygroscopic absorption of water by the material, thus influencing optical properties [33].

The abrasive systems in dentifrices must have sufficient abrasiveness to clean teeth effectively. However, the level of abrasion on the enamel and dentin should avoid damaging the teeth and the restorative materials’ surface during regular use. The cosmetic restorative materials’ surface conditions play a crucial role in their color stability. Fine-color particles can become trapped in the material’s pits, leading to surface adsorption-induced discoloration, which can be prevented through toothbrushing. In this study, it was assumed that brushing the specimens prevented the colorants from adhering to the material’s surface, thus reducing the amount of color change over time. Da Costa et al. [12] reported, on an estimated basis, that each tooth might receive 8 s of brushing per day. Therefore, being a 1-month simulation, a total of 240 s of brushing were performed in groups 3 and 4, equally distributed between the staining cycles.

The RDA values in dentifrices can differ depending on the formula used, typically falling between 30 and 200–250, the latter being the American Dental Association recommended limit [34].

Significant differences were reported in terms of color change between G3 and G4. However, since the only difference between G3 and G4 was the RDA of the toothpastes (30 and 90, respectively), it can be affirmed that RDA is responsible for different color change. Conversely, no significant differences in translucency have been reported between G3 and G4 after staining. Therefore, the second null hypothesis has to be rejected since toothpaste with different RDA produces significant changes in translucency.

In particular, the lowest color change after staining (T1) was observed for G3, where a low-abrasive toothpaste was used. Although it may be considered that strong abrasive toothpastes (G4) may reduce RBC staining, they behaved like the group in which specimens were only cycled between staining solution and distilled water (G2).

This study’s results agree with Torso et al. [22], who reported that specimens brushed with very strong abrasives showed high ΔE_00_ values (higher staining) with respect to specimens brushed with lower RDA toothpastes. It may therefore be speculated that higher abrasion can occur with strong abrasive toothpaste and that higher pigments’ adsorption may be related to a rougher surface.

Since staining depends on the toothpaste’s abrasion level, our findings partially agree with several other papers that found that regular brushing can decrease or even prevent staining from coffee and tea on resin-based veneering materials [13,30].

In our study, an electric toothbrush has been chosen. This type of toothbrush is easier to use in an experimental set-up since it is characterized by its movement and is exempt from stroke simulations. In addition, a brushing force of 200 g was used, which falls within the mid-range of the minimum and the maximum allowed by ISO standards for laboratory testing [35,36,37].

This study simulated a 1-month exposure to a staining solution with or without brushing simulation. Although brushing simulation can reduce color change, it should be reminded that extrinsic staining accumulates and is exacerbated by the degradation of the material. Hence, as the aging time increases, it becomes less likely that brushing and polishing can avoid the discoloration of resin composites [8,38]. Future studies could be conducted to investigate the effect of brushing simulation on aged composites.

Discoloration may or not be clinically detectable [25]. Significant differences in color parameters, in fact, may or may not have a clinical consequence. Some color changes can in fact be detectable by scientific tools (such as spectrophotometers), but an observer could not detect any difference. In this case, we are talking of a color change below the PT. If the color change is detectable but still clinically acceptable, the color change is between the PT and the AT. If the color change is not clinically acceptable, it is over the AT [39]. In our study, G3 was below the AT, suggesting that brushing the specimen with a toothpaste with low RDA (30) was able to maintain color within the clinical acceptability threshold. The very strong abrasive toothpaste could leave a rougher surface more susceptible to staining. Conversely, the low-abrasion toothpaste could act as a polishing media, removing superficial staining and keeping the surface smooth.

Repolishing produced significant changes in color and translucency. Therefore, hypothesis #3 has to be rejected. Regardless of significant color change, repolishing reverted color change below the AT. After repolishing, all groups showed a perceptible but clinically acceptable color change (>ΔE00 = 0.81 <ΔE00 = 1.77) [25]. From a clinical point of view, this finding suggests that in some clinical situation, repolishing stained restorations could prevent an immediate substitution of the restoration, increasing the survival rate [32].

Translucency refers to a material’s ability to allow partial transmission of light while scattering it so that objects beneath it cannot be seen [40]. The translucency of RBCs can be impacted by factors such as the polymeric matrix, filler particles, pigments, and layer thickness [41,42,43]. In our study, TP values of the brushing groups were significantly lower than the other groups, suggesting that brushing could raise specimen’s opacity. This finding could be related to the change in roughness induced by the brushing simulation since a material-dependent influence of surface roughness on translucency has been reported [44]. Repolishing procedures were able to revert specimens’ translucency, but a full recovery was not possible. This finding could be related to the fact that repolishing could only remove a superficial layer, while a deeper penetration of the staining substances could have occurred. Among the limitations of this study are that a single type of resin-based composite and a single staining liquid were used. This choice was made to keep these factors as constants while focusing on evaluating the contribution of the other investigated procedures. The findings of this study can be further investigated including an analysis of factors such as bonding systems and bonding between materials as suggested in other studies [45]. Furthermore, other staining liquids such as other beverages [1] or mouth rinses [46] shall be included. Further studies are needed to determine a reliable, standardized experimental design able to simulate the natural clinical scenario as much as possible.

## 5. Conclusions

Within the limitations of the current study, it can be concluded that the color stability and translucency of a nano-hybrid RBC is influenced by the staining procedure and toothpaste’s RDA. Repolishing was able to partially recover the color and translucency changes.

## Figures and Tables

**Table 1:** Composition of the nano-hybrid resin-based composite.

Nano-Hybrid Composite	Manufacturer	wt%/vol%	Matrix	Filler	Particle Size Range	Lot.
Clearfil Majesty Classic ES-2 (A2)	Kurary Noritake Dental Inc., Okayama, Japan.	78%/40%	Bis-GMA Hydrophobic aromatic dimethacrylate dl-Camphorquinone	Silanated barium glass filler Prepolymerized organic fillers	0.37 to 1.5 μm	270028

Abbreviations: Bis-GMA (Bisphenol A-diglycidyl dimethacrylate).

**Table 2:** Values at baseline.

	Groups							
	1		2		3		4	
	Mean	sd	Mean	sd	Mean	sd	Mean	sd
TP	7.77	0.62	8.05	0.67	8.26	0.47	8.03	0.87
CR	0.87	0.01	0.86	0.02	0.86	0.01	0.86	0.02

**Table 3:** TP Parameter: values for each timepoint and group adjusted for baseline values.

	Groups							
Time	1		2		3		4	
	Mean	SE	Mean	SE	Mean	SE	Mean	SE
0	8.01	0.11	8.03	0.11	8.04	0.11	8.03	0.11
1 ^@ + ° ^ *^	7.36	0.11	7.96	0.11	6.77	0.11	6.88	0.11
2 ^# °^	6.88	0.11	7.06	0.11	7.13	0.11	6.69	0.11

Mean and se estimated from mixed model adjusted for baseline values. BH corrections: ^ differences G1 vs. G3; * differences G1 vs. G4; ° differences G2 vs. G4; @ differences G2 vs. G1; + differences G2 vs. G3, # differences G3 vs. G4.

**Table 4:** Estimated mean from mixed model corrected by baseline values.

	Groups							
Time	1		2		3		4	
	Mean	SE	Mean	SE	Mean	SE	Mean	SE
0	0.86	0.004	0.86	0.004	0.86	0.004	0.86	0.004
1	0.87	0.004	0.86	0.004	0.88	0.004	0.88	0.004
2 ^* ° #^	0.88	0.004	0.88	0.004	0.88	0.004	0.89	0.004

Mean and se estimated from mixed model adjusted for baseline values. BH corrections* differences G1 vs. G4; °differences G2 vs. G4; # differences G3 vs. G4.

**Table 5:** ΔE00 Parameter: estimated means and SE from mixed model.

	Groups							
Time	1		2		3		4	
	Mean	SE	Mean	SE	Mean	SE	Mean	SE
1 ^^ * @ + #^	2.65	0.05	2.22	0.06	1.66	0.06	2.19	0.07
2 ^@ ^ * +^	1.38	0.05	1.19	0.06	0.93	0.06	1.12	0.07

Mean and se estimated from mixed model. BH corrections: ^ differences Group 1 vs. Group 3; * differences Group 1 vs. Group 4, @ differences Group 2 vs. Group 1; + differences Group 2 vs. Group 3, # differences Group 3 vs. Group 4.

## Data Availability

No new data were created.
